# Using Portable Ultrasound to Monitor the Neuromuscular Reactivity to Low-Frequency Electrical Stimulation

**DOI:** 10.3390/diagnostics11010065

**Published:** 2021-01-03

**Authors:** Alin Petraş, Răzvan Gabriel Drăgoi, Vasile Pupazan, Mihai Drăgoi, Daniel Popa, Adrian Neagu

**Affiliations:** 1 Department of Medical Rehabilitation, Balneology and Rheumatology, Victor Babeş University of Medicine and Pharmacy Timişoara, 300041 Timişoara, Romania; alinpetras1991@gmail.com (A.P.); dragoi.mihai@umft.ro (M.D.); popa.daniel@umft.ro (D.P.); 2 Department of Functional Sciences, Victor Babeş University of Medicine and Pharmacy Timişoara, 300041Timişoara, Romania; pupazan.vasile@umft.ro (V.P.); neagu@umft.ro (A.N.); 3 Department of Physics and Astronomy, University of Missouri, Columbia, MO 65211, USA

**Keywords:** M-mode scan, muscle activation, Teager–Kaiser energy operator (TKEO), electrotherapy, sensory threshold, motor threshold

## Abstract

Neuromuscular electrical stimulation (NMES) is useful for muscle strengthening and for motor restoration of stroke patients. Using a portable ultrasound instrument, we developed an M-mode imaging protocol to visualize contractions elicited by NMES in the quadriceps muscle group. To quantify muscle activation, we performed digital image processing based on the Teager–Kaiser energy operator. The proposed method was applied for 35 voluntary patients (18 women and 17 men), of 63.8 ± 14.1 years and body mass index (BMI) 30.2 ± 6.70 kg/m^2^ (mean ± standard deviation). Biphasic, rectangular electric pulses of 350 µs duration were applied at two frequencies (60 Hz and 120 Hz), and ultrasound was used to assess the sensory threshold (ST) and motor threshold (MT) amplitude of the NMES signal. The MT was 23.4 ± 4.94 mA, whereas the MT to ST ratio was 2.69 ± 0.57. Linear regression analysis revealed that MT correlates poorly with body mass index (R^2^ = 0.004) or with the thickness of the subcutaneous adipose tissue layer that covers the treated muscle (R^2^ = 0.013). Our work suggests that ultrasound is suitable to visualize neuromuscular reactivity during electrotherapy. The proposed method can be used in the clinic, enabling the physiotherapist to establish personalized treatment parameters.

## 1. Introduction

Electrical stimulation for muscle strengthening is clinically useful in cases of immobilization, in early rehabilitation and in muscle reeducation [1]. Neuromuscular electrical stimulation (NMES) is especially useful when the patient is incapable of intense voluntary exercise [1]. NMES differs from voluntary exercise in three main ways [2]. First, electrical stimulation recruits motor units in a reversed order because excitability is greater for motor neurons whose axons are larger in diameter. Second, electrical stimulation results in the firing of all motor neurons within the stimulated region. Third, sensory nerves are inevitably stimulated, as are pain fibers [2].

Both electrical stimulation and voluntary strength training are able to increase the force of maximal voluntary contraction, albeit through different mechanisms [3]. A systematic review of 35 randomized controlled trials demonstrated that NMES is less effective than volitional exercise, but it is significantly better than no exercise [4]. Electrical stimulation is not recommended as a substitute for physical activity, but it is useful during immobilization and in conditions that interfere with voluntary training.

Electrotherapy might be ineffective if muscle response is not tested. A weak effect of NMES [5] might result if the amplitude of the applied electric signal is too low [4]. Indeed, Sisk et al. relied on a subjective criterion, increasing the current output until “a visible contraction” was achieved [5]. Objective tracking of the neuromuscular reactivity is of utmost importance in cases of muscle reeducation, when the recommendation is to use the weakest electric signal that triggers a muscle contraction [6].

Rehabilitative ultrasound imaging (RUSI) is a rapidly evolving research field that aims to investigate the morphology and function of muscles and related soft tissues [7,8]. The validity of RUSI was tested against criterion methods, such as magnetic resonance imaging and computed tomography, in the general population [7] as well as in the elderly [9]. Several studies evaluated the reliability of RUSI, with intraclass correlation coefficients (ICC) > 0.72—the highest for the vastus lateralis muscle (ICC ranging from 0.852 to 0.999) [9]. Muscle contraction was quantified via ultrasound(US) imaging and related to muscle activity described by electromyography (EMG) over a full range of isometric contractions [10].

Dynamic US imaging was used in combination with speckle tracking to visualize and quantify muscle contraction. This technique enables one to spot even slight muscle contractions [11,12]. Although effective, dynamic US imaging requires expensive equipment and specially trained personnel.

This work is motivated by the need to track the effectiveness of NMES in real time, in a typical bedside environment. Muscle activation can be assessed by dynamometry and EMG [10], but these methods have limitations in the clinical context. Dynamometry requires a specific experimental setup for each muscle group and can be applied only in the absence of immobilization or pain related to muscle contraction. EMG cannot be applied simultaneously with electrical stimulation. It is suitable for evaluating treatment results, but its accuracy depends on the clinician’s skill. Here, we propose a methodology for monitoring the neuromuscular response to NMES using a portable US instrument originally developed for body composition assessment [13,14]. Besides being affordable, the chosen instrument is appealing because of its user-friendly software.

## 2. Materials and Methods 

### 2.1. Patients

Participating subjects were recruited from the pool of patients treated at the Clinic of Medical Rehabilitation, Balneology and Rheumatology from Timișoara, Romania. Patients gave written informed consent to be included in this investigation. This study was conducted in accord with the Declaration of Helsinki and it was approved by the Committee of Research Ethics of the “Victor Babeș” University of Medicine and Pharmacy Timișoara (32/11.04.2019).

To be enrolled, potential subjects were required to meet all of the following *inclusion criteria:* (i) atrophy of the quadriceps femoris muscle group due to affections of the hip joint and/or knee joint caused by injuries, osteoarthritis or other rheumatic condition, (ii) inability to perform high-intensity volitional exercise evaluated by a senior kinesiologist (>20 years of experience), (iii) existence of a physician’s referral to participate in a NMES program for the strengthening of the quadriceps femoris, (iv) age 18–85 years, and (v) willingness and ability to provide written informed consent. A potential subject was deemed ineligible for this study if she/he met one or more of the following *exclusion criteria*: (i) denervated quadriceps femoris muscles, (ii) hemiplegia after stroke, (iii) impaired lower limb function caused by spinal cord injury or multiple sclerosis, (iv) spasticity inflicted by a neurological condition, and (v) rupture of the patellar tendon or quadriceps tendon.

A total of 35 patients (18 women and 17 men) were enrolled in this study, as described in Table 1 in terms of mean value ± standard deviation (SD) and the range of values. The body mass index (BMI) describes the patient’s nutritional status; it is defined as body mass (kg) divided by height squared (m^2^). Spanning a wide range of BMIs, the investigated sample enabled us to evaluate correlations between nutritional status and electrical stimulation parameters.

### 2.2. Electrotherapy Monitored via Ultrasound Imaging

Each patient was treated, for about 20 min, with biphasic rectangular electric pulses delivered by a Gymna Combi 400 physiotherapy instrument (GymnaUniphy, Bilzen, Belgium). The pulse duration was 350 µs, with an interphase interval of 100 µs. The pulse amplitude was raised gradually from 0 mA to the desired value during a 2 s surge stage. The amplitude was maintained at the desired value for 12 s and it was reduced to 0 mA during a 2 s shrink stage. A pause of 12 s was kept between successive stimulations. Electrical stimulation was administered at two different pulse frequencies (60 and 120 Hz).

Electric pulses were applied in a bipolar configuration, using 7 × 4 cm carbon rubber electrodes wrapped in Chamex sponge bags soaked in physiological saline (sterile aqueous solution of 0.9% NaCl). We placed the electrodes with their short side oriented along the longitudinal axis of the rectus femoris muscle and their centers located at a distance of 10 cm from the nearest bony anatomical landmark employed in anthropometry (trochanterion for the proximal electrode and anterior patella for the distal electrode [15]).

We covered the ultrasound probe with about 0.25 mL ultrasound transmission gel and placed it perpendicularly to the skin, midway between the two electrodes—a portion of the thigh hereafter referred to as region of interest. The ultrasound probe was maintained in a steady position during image acquisition. The ultrasound instrument, BodyMetrix™ BX2000 (IntelaMetrix, Livermore, CA, USA) was controlled by the BodyView™ software v5.7.11043 (IntelaMetrix, Livermore, CA, USA) [13].

For each patient, we created a profile, specifying name, birth date, gender, height, weight, body constitution (obese, non-athletic, athletic, or elite). First, we clicked the “Measure” tab and performed one measurement of the thickness of the subcutaneous fat layer covering the quadriceps muscle group in the region of interest. Then, the instrument was switched to “Scan” mode by clicking the corresponding tab in BodyView. Finally, we selected “Thigh” from the “Scan Point” drop-down list, and set the “Maximum Depth” slider to 60 or 100 mm, depending on the patient’s body constitution.

Image acquisition commenced prior to the surge stage of electrical stimulation and continued during the stimulation sequence beyond the end of the shrink phase. The recorded image appeared in the “Tissue Cross-Section” panel of BodyView. The recorded images were visualized and exported in portable document format (PDF) using the “Compare Images” button. Adobe Acrobat 9 Pro (Adobe, San Jose, CA, USA) was used to convert the images to TIFF format setting the resolution to 1200 dots per inch. 

The BodyView software does not associate a time axis to the scan plots. Here, we relied on the timer of the electrotherapy instrument: we commenced the scan at the middle of a pause and proceeded until the middle of the next pause, ensuring a scan duration of about 28 s (6 s pause, 2 s surge, 12 s of electrical stimulation at full amplitude, 2 s shrink, and 6 s pause). Dividing the width of the scan image by 28, we found that a one-second recording comprised about 100 pixels along the horizontal direction.

For each subject, we saved and analyzed three scan images before electrical stimulation and three images for each combination of NMES amplitude (sensory threshold (ST) and motor threshold (MT)) and pulse frequency (60 and 120 Hz). ST is a subjective parameter, defined as the smallest amplitude of the NMES current intensity that elicits a sensory response in the patient. By contrast, MT is an objective quantity, defined as the smallest amplitude of the NMES current intensity that triggers a muscle contraction strong enough to be revealed by US imaging.

### 2.3. Data Analysis

Experimental data were analyzed using MATLAB 7.13 (The MathWorks, Inc., Natick, MA, USA). Normal distribution was assessed using the Jarque–Bera test [16]. Student’s two-sample *t*-test was employed to compare the means of two independent datasets. Statistical significance was set at *p* ≤ 0.05.

Digital image processing was done using the Teager–Kaiser energy operator (TKEO) along the lines developed by Dieterich et al. [17]. For a discrete-time signal, *x*(*n*), where *n* is a natural number that labels successive time points, the output of the TKEO is given by [18]: *ψ*_d_[*x*(*n*)] = *x*^2^(*n*) − *x*(*n* − 1)·*x*(*n* + 1). First, the region of interest was cropped from the M-mode US trace; it was converted to a grayscale image and the TKEO output was computed for the pixel values, *x*(*n*), of each row of pixels. Then, for each instant of time (each *n*), we computed the mean value and standard deviation (SD) of the TKEO output values obtained for all pixel lines. To smooth the data, we applied a moving average filter with a span that encompassed data points recorded in 0.3 s. We analyzed the plots of these quantities vs. time to evaluate the muscle response to electrical stimulation.

We used M-mode US imaging to assess the muscle reactivity to NMES. To evaluate the hypothesis that certain NMES parameters depend on the nutritional status of the patient, we performed a linear regression analysis of these quantities versus the patient’s BMI and versus the thickness of the subcutaneous adipose tissue (SAT) layer that covered the patient’s quadriceps femoris muscles at the region of interest.

## 3. Results

### 3.1. M-mode Ultrasound Traces Recorded during Electrotherapy

**Figure 1:**
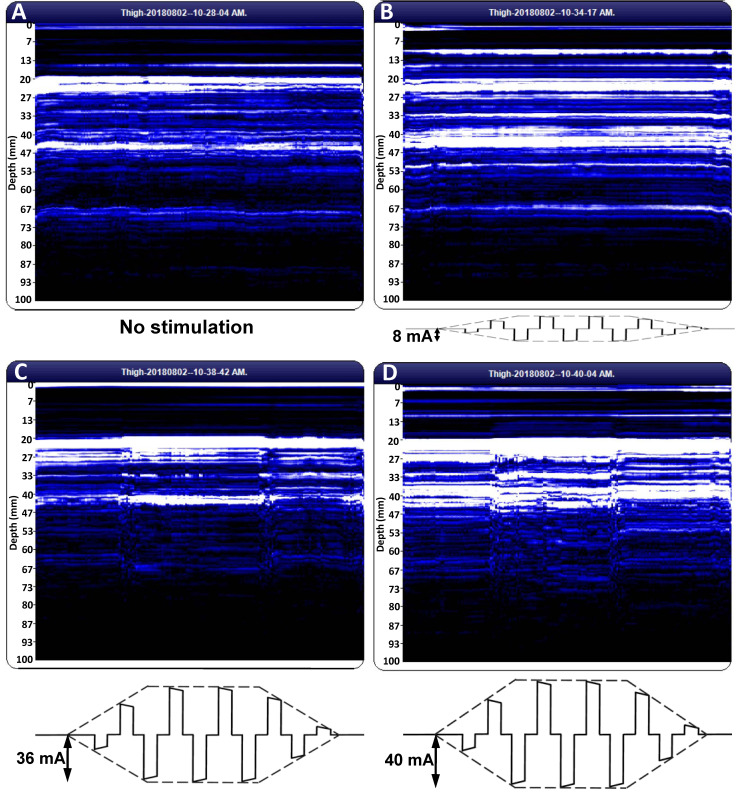
M-mode ultrasound (US) images acquired during neuromuscular electrical stimulation (NMES). US scans of the quadriceps muscle group recorded in the absence (**A**) and in the presence (**B**–**D**) of NMES, with the amplitude of the rectangular pulses set to the sensory threshold (ST) (**B**), the motor threshold (MT) (**C**), and 10% above the MT (**D**). During the scan, the US transducer was maintained in a steady position, midway between the NMES electrodes.

We used the BodyMetrix device in scanning mode to visualize tissue interfaces beneath the skin during the NMES treatment of quadriceps femoris. Figure 1 represents these interfaces in the absence of electrical stimulation (A), in the presence of the weakest perceptible electric stimulation (B), in the presence of the weakest electrical stimulation able to cause a motor response (C), and for a 10% increment over the MT (D). A 10% decrement of the amplitude of the rectangular electric pulse, on the other hand, caused a cessation of the muscle contraction, the recorded image being similar to Figure 1A,B (not shown).

In the M-mode US scans of Figure 1, each line of pixels describes a muscle layer of 0.042 mm in thickness situated at a given depth beneath the skin. Two successive pixels are recorded 10 ms apart. If the muscle is at rest, more echogenic (connective tissue) layers appear as bright horizontal stripes on the M-mode image; these are separated by dark stripes corresponding to less echogenic (contractile tissue) layers. As muscle contraction proceeds, the path of the US beam is crossed by tissue layers of various echogenicity, causing differences in brightness between successive pixels corresponding to a given depth [17]. This change in the pattern of the M-mode US trace was found to be a reliable indicator of the onset of muscle contraction [17,19]. Visual inspection of an M-mode scan is suitable to ascertain whether a muscle contraction occurs or not, but digital image processing is needed to assess the extent of contraction.

**Figure 2:**
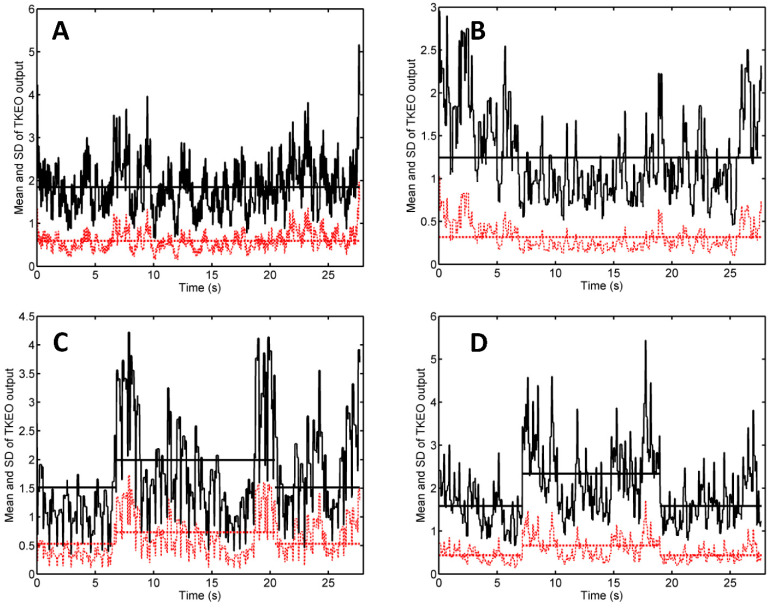
Teager-Kaiser energy operator (TKEO) analysis of the M-mode US scans shown in Figure 1. Panels (**A**–**D**) correspond to the respective panels of Figure 1. Portions of the M-mode US scans corresponding to the depth range of 2–6 cm have been cropped, converted to grayscale, and the resulting pixel values were transformed via the TKEO operator. At any instant of time, the mean value and standard deviation (SD) of the TKEO output were computed and plotted as a red dashed line and black solid line, respectively.

Shown in Figure 2 are the results of the quantitative analysis of the US scans of Figure 1. For each scan, the portion of the image corresponding to depths ranging from 2 to 6 cm has been cropped, converted to grayscale, and transformed, line-by-line, using the TKEO. We computed the mean value and the standard deviation (SD) of the TKEO outputs of all the pixel lines from the image [17]. We used a moving average filter with a span of 31 time points to smooth the data and plotted the time dependence of the mean value of the TKEO outputs (dashed-dotted line) and their SD (solid line) (Figure 2). The latter describes the heterogeneity of the TKEO outputs corresponding to various depths and has been proposed as an indicator of muscle contraction [17].

In the absence of electrical stimulation (Figure 2A), or at the ST (Figure 2B), both TKEO signals suffer random fluctuations around their respective time averaged values, represented as horizontal lines. When electrical simulation is turned on at the MT level (Figure 2C) or above (Figure 2D), the TKEO signals increase suddenly.

The Jarque–Bera test indicated that the data were normally distributed. In the context of Figure 2C, the mean TKEO output had a time average of 0.53 in the absence of stimulation and 0.73 in the presence of MT level NMES; the 0.2 increment was statistically significant according to a two-sample *t*-test (*p* < 0.001), with a 95% confidence interval (CI) for the true difference of population means of [0.17, 0.22]. For the SD of TKEO outputs, the time average was 1.51 in the absence of stimulation and suffered a statistically significant (*p* < 0.001) change of 0.44, with 95% CI [0.42, 0.54]. For the data of Figure 2D, the increment of the time average of the mean TKEO output was 0.23, with 95% CI [0.21, 0.25], whereas for the SD of TKEO outputs it was 0.76, with 95% CI [0.70, 0.80].

**Figure 3:**
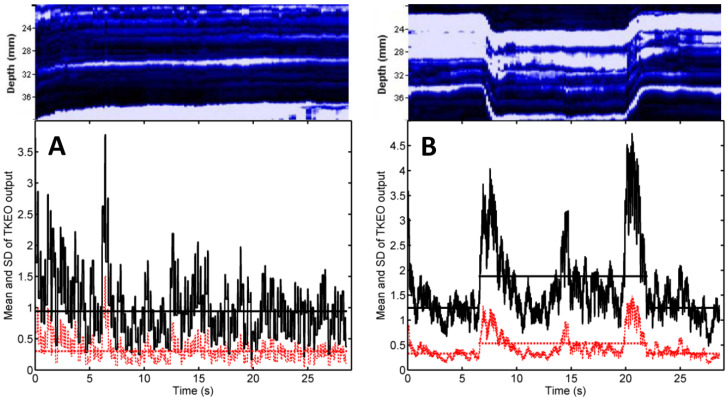
Representative M-mode US scans and their TKEO analysis. The shown images are scan fragments that correspond to the depth range 20–40 mm beneath the skin, recorded while the quadriceps was not stimulated (**A**) and in the presence of NMES at the MT amplitude of 24 mA (**B**). Notations are explained in the caption of Figure 2.

The scans shown in Figure 1 and analyzed in Figure 2 refer to the male patient with the highest MT from the investigated sample (36 mA). Figure 3 shows representative results corresponding to another male patient, whose MT is 24 mA—close to the sample mean (see Table 2).

The mean TKEO output, plotted as a red dashed line, had roughly the same time average (red dashed horizontal line) when the muscle was not stimulated (Figure 3A), and in the pause before and after electrical stimulation (Figure 3B, first and last 6 s). NMES caused a statistically significant (*p* < 0.001), 61% increase in the time average of the mean TKEO output, from 0.33 to 0.53. The SD of the TKEO output (black solid line) had a time average of 1.25 during the pause and increased significantly due to NMES, reaching 1.88 (Figure 3B, black solid horizontal lines).

### 3.2. Parameters of Neuromuscular Electrical Stimulation

We used the above imaging protocol to estimate the MT. To this end, we raised the signal amplitude until a sensory response was reported by the patient (i.e., until we reached ST), and started the evaluation of the MT at twice the ST [2]. If muscle response was absent at this level, we increased the signal amplitude gradually, in 1 mA steps, until muscle contraction was apparent on the M-mode US trace and confirmed by TKEO analysis (see Figure 1C and Figure 2C). If muscle contraction was already observed at twice the ST, we lowered the signal amplitude, in 1 mA steps, until the contraction ceased, and identified the MT as the smallest NMES amplitude able to trigger muscle response. Once ST and MT were evaluated at an NMES signal frequency of 60 Hz, we switched to 120 Hz, but we found the same ST and MT at both frequencies.

Table 2 presents the descriptive statistics of the MT amplitude of the NMES pulse, the ST amplitude of the NMES pulse and their ratio (MT to ST ratio).

According to the *p*-values of the Jarque–Bera test (Table 2, last column), the MT values are normally distributed (*p* > 0.05), whereas the ST values are not; the MT to ST ratio deviates marginally from a normal distribution. 

**Figure 4:**
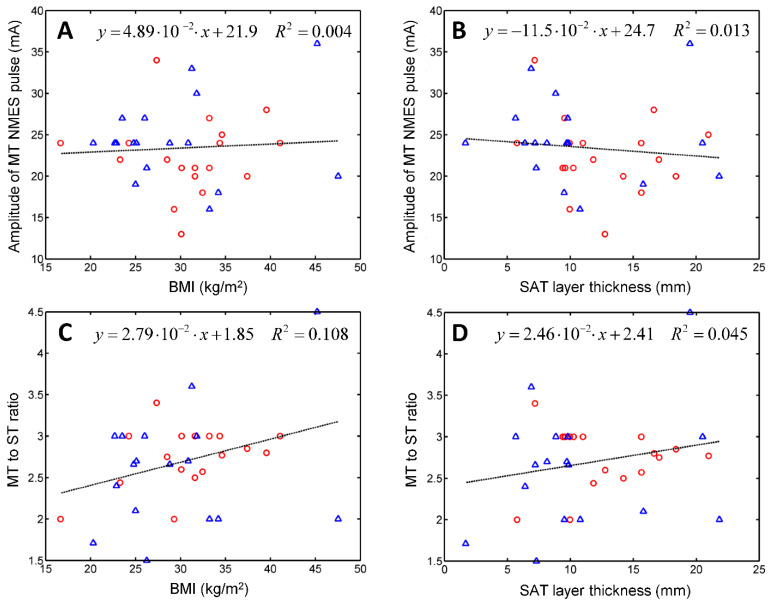
Scatter plots of the amplitude of the biphasic rectangular electric pulses of 0.35 ms duration needed to trigger the contraction of the quadriceps muscle vs. the BMI of the patient (**A**) and vs. the thickness of the subcutaneous adipose tissue (SAT) layer in the treated region (**B**), and the MT to ST ratio vs. BMI (**C**) and the SAT layer thickness (**D**). Circles and triangles represent data points of women and men, respectively.

Figure 4 represents the scatter plots and linear regression parameters of MT vs. BMI (A) and SAT layer thickness (B), and the MT to ST ratio vs. BMI (C) and SAT layer thickness (D).

Figure 4 indicates that the NMES treatment parameters slightly correlate with the nutritional status of the patient. The coefficients of determination range from 0.004 to 0.108.

## 4. Discussion

Using an instrument originally developed for body composition assessment via A-mode US [14], we developed an M-mode US-imaging protocol that enabled the physiotherapist to visualize muscle fasciculation contractions triggered by electrical stimulation. The proposed method requires a portable and affordable instrument, which is bundled with a user-friendly software [13]. Our study aimed to demonstrate that US is suitable for ascertaining that the treated muscle responds to the NMES signal.

Why should we track the motor response during a NMES treatment? Because the efficacy of NMES depends on the elicited response and its subjective assessment is unreliable [4]. There is no standard method for the evaluation of muscle response to NMES. Surface EMG and dynamometry are commonly used for this purpose [20,21], but they are difficult to implement in a routine clinical setting.

Our work is not the first to use M-mode US for detecting muscle activation. Vasseljen et al. investigated the timing of the onset of the lumbar multifidus muscle activity by digital image processing of M-mode US scans via root mean square calculations, and tested their approach against intramuscular EMG [22]. Previously, B-mode and M-mode US were used to measure changes in the thickness of the transversus abdominis muscle during maximum voluntary contractions associated with the low abdominal hollowing manoeuvre [23]. A positive correlation was found between the EMG signal and the change in muscle thickness (*R*^2^ = 0.87). The reliability of US measurements was high: ICC was 0.989 for B-mode and 0.981 for M-mode [23]. RUSI measurements of muscle thickness, however, might not detect the activation of certain muscles, presumably because competing forces exerted by surrounding muscles hamper the thickening of the muscle of interest [7]. For example, Hodges et al. observed a change in thickness due to activation in transversus abdominis but not in the obliquus externus [10].

Although the instrument used in our study enables one to measure subcutaneous tissue layer thickness, and compares well in accuracy with high-resolution B-mode US [24], here we did not evaluate muscle activation by measuring changes in muscle thickness. Instead, we relied on digital image processing based on the Teager–Kaiser energy operator to quantify changes in the pattern of M-mode US scans associated with muscle activation. This approach, proposed by Dieterich et al., has been validated by comparison with fine-wire EMG [17] and applied in patients with chronic hip pain [19].

In our TKEO analysis, the mean Teager–Kaiser energy (TKE) displayed a pair of narrow peaks at the onset and cessation of the muscle contraction (Figure 2C,D). By contrast, voluntary contractions of gluteus minimus were associated with less abrupt changes and wider peaks of the mean TKE and its SD [17]. This discrepancy reflects the differences between muscle activation patterns associated with electrical stimulation and volitional exercise: NMES triggers the simultaneous activation of all motor units from the stimulated region, whereas a voluntary contraction proceeds by gradual recruitment of motor units [2].

The narrow peaks of the mean TKEO signals observed when electrical stimulation was turned on and off enable the user to avoid false positive (type I) errors. In the study of Dieterich et al. on the voluntary activation of hip abductors, M-mode US occasionally indicated muscle motion before EMG onset (i.e., before excitation) [17]. Such type I errors were attributed to passive muscle motion occurred as the muscle’s connective tissue was pulled by earlier activating motor units from the monitored muscle’s neighborhood. In the context of electrical stimulation, neighboring muscles are less likely to become activated than the muscle situated closest to the pair of NMES electrodes. Transducer motion can also produce changes in the texture of US scans. Such motion artifacts were observed in our study (see, e.g., Figure 2B, first 3 s and last 2 s, and Figure 3A, first 7 s) but they did not occur in synchrony with the start and the end of the electrical stimulation. Hence, our findings suggest that false positive errors are unlikely when M-mode US is used to monitor muscle activation during NMES. Nevertheless, motion artifacts can lead to false negative (type II) errors. They boost the time average of the TKEO output recorded in the absence of stimulation (see, e.g., Figure 2C,D, first 6 s and last 6 s). Even if the muscle responds to the electrical stimulation, the difference between the time averages of the TKEO signals recorded in the presence and in the absence of NMES might not reach statistical significance. Therefore, according to the criterion used in the present study, the NMES signal would be deemed ineffective. Further investigations will be needed to minimize transducer motion and/or to eliminate its contribution to the TKEO signal, as well as to devise better criteria for quantifying the extent of muscle activation based on M-mode US imaging. 

We employed our US imaging protocol to assess ST and MT, and test their correlations with the subject’s BMI or with the thickness of the subcutaneous adipose tissue (SAT) layer that covers the quadriceps. SAT layer thickness was found to affect the efficacy of NMES [25,26]. Miller et al. divided their sample into skinfold thickness (SFT) tertiles and measured the maximum comfortable NMES signal amplitude and the corresponding torque [26]. ANOVA revealed significant differences between the mean NMES amplitudes tolerated by subjects from the lower and upper tertiles. A positive correlation was found between NMES amplitude and SFT (*R*^2^ = 0.31). For NMES force production, the effect of SFT category was not significant [26]. Medeiros et al. observed a positive correlation between the maximal NMES signal and SFT and a negative correlation between NMES-evoked torque and SFT [25]. No correlation was reported between the central activation ratio and the thickness of the subcutaneous fat layer [27]. Sensory detection of hot and cold was also found to correlate with the amount of body fat characterized in terms of the BMI [28] and SAT thickness [29]. Therefore, we hypothesized that ST and MT might depend on the nutritional status of the patient. Linear regression analysis revealed that electrical stimulation parameters correlate poorly with nutritional status; neither BMI nor SAT thickness were good predictors of ST or MT.

Our results are consistent with the literature: a perceptible sensation was reported to result from electric pulse amplitudes of 2–10 mA, whereas muscle contraction required 20–50 mA [30,31]. For muscle strengthening, the therapist may opt for pulse amplitudes of 30–50 mA, if the patient tolerates them. It is recommended to track muscle reactivity when one seeks to minimize discomfort and maximize the spatial recruitment of motor units [31]. Monitoring the neuromuscular response to electrotherapy is important when the treatment aims at the motor restoration of stroke patients [6]. In these patients, pulse amplitudes at or above the MT induce sensory-mediated modifications in central motor pathways that help with restoring the intrinsic functional capacity [32].

Limitations: The relatively small sample size is a limitation of this study. To assess the reliability of ultrasound for monitoring the effectiveness of NMES, further investigations should be performed on larger and more diverse cohorts.

The image analysis procedure should be improved by devising filters to eliminate transducer motion artifacts apparent at the beginning and the end of the recording (Figure 1).

Another limitation of this work is related to the treated region. The generous size of the quadriceps muscle group and the easily palpable anatomic landmarks that delimit the thigh facilitated our protocol. Further research will be needed to implement the proposed methodology for smaller muscles located in more complex anatomical neighborhoods.

Strengths and clinical implications: The present study demonstrates that NMES treatment parameters differ from one patient to another and correlate poorly with nutritional status. Assessing the response to electrotherapy is necessary when the treatment protocol calls for the smallest pulse amplitude able to cause a muscle contraction. M-mode US imaging is an appealing option for this purpose because it is noninvasive, it can detect muscle activation, and it can be applied during electrical stimulation performed in a clinical practice.

## 5. Conclusions

This work aimed at evaluating the hypothesis that ultrasound can be used in the clinic to ascertain the efficacy of NMES. To this end, M-mode ultrasound scans of quadriceps femoris were recorded simultaneously with electrical stimulation. Starting from a certain level of the NMES signal amplitude (the motor threshold), muscle activation was detected as a change in the texture of the recorded picture. Image analysis based on the Teager–Kaiser energy operator enabled a quantitative assessment of the muscle contraction elicited by electrical stimulation. Our study suggests that M-mode ultrasound imaging may help to determine personalized NMES treatment parameters.

## Figures and Tables

**Table 1:** Characteristics of the study group.

	All (*n* = 35)		Women (*n* = 18)		Men (*n* = 17)	
	Mean ± SD	Range	Mean ± SD	Range	Mean ± SD	Range
Age (y)	63.8 ± 14.1	(25, 84)	68.2 ± 7.8	(59, 82)	59.1 ± 17.7	(25, 84)
Height (m)	1.66 ± 0.10	(1.48, 1.87)	1.59 ± 0.06	(1.48, 1.70)	1.74 ± 0.07	(1.60, 1.87)
BMI (kg/m^2^)	30.2 ± 6.7	(16.7, 47.5)	31.0 ± 5.9	(16.7, 41.1)	29.4 ± 7.6	(20.3, 47.5)

**Table 2:** Descriptive statistics of the MT, ST, and the MT to ST ratio, computed for *n* = 35 subjects (18 women and 17 men). Shown are the mean value, standard deviation (SD), standard error (SE), range (min., max.), skewness, kurtosis and the *p*-value of the Jarque–Bera test.

Quantity	Mean	SD	SE	Range	Skewness *	Kurtosis *	*p* *
MT (mA)	23.4	4.94	0.84	(13, 36)	0.52	3.52	0.191
ST (mA)	8.86	1.78	0.30	(5, 14)	1.13	5.35	0.006
MT to ST ratio	2.69	0.57	0.10	(1.5, 4.5)	0.45	4.59	0.046

* These quantities are dimensionless. For a normal distribution, *p* > 0.05, skewness = 0, and kurtosis = 3.

## Data Availability

The data related to this study are available upon request from the corresponding author, R.G.D., with the exception of raw data items that could compromise the privacy of study participants.

## Abbreviations

BMI	body mass index
CI	confidence interval
EMG	electromyography
ICC	intraclass correlation coefficient
MT	motor threshold
NMES	neuromuscular electrical stimulation
RUSI	rehabilitative ultrasound imaging
ST	sensory threshold
SFT	skinfold thickness
SD	standard deviation
SAT	subcutaneous adipose tissue
TKEO	Teager-Kaiser energy operator
US	ultrasound
